# Unlike severe periodontitis, caries does not associate with intracranial aneurysms or aneurysmal subarachnoid hemorrhage

**DOI:** 10.1007/s00701-022-05406-4

**Published:** 2022-11-23

**Authors:** Joona Hallikainen, Tanja Pessi, Miira Vehkalahti, Anna Liisa Suominen, Mikko Pyysalo, Juhana Frösen

**Affiliations:** 1 Hemorrhagic Brain Pathology Research Group, University of Tampere, Tampere, Finland; 2 Oral and Maxillofacial Diseases, Kuopio University Hospital, Kuopio, Finland; 3 Oral and Maxillofacial Diseases, Central Finland Central Hospital, Helsinki, Finland; 4 Oral and Maxillofacial Diseases, University of Helsinki, Helsinki, Finland; 5 Finnish Institute for Health and Welfare, Helsinki, Finland; 6 Institute of Dentistry, University of Eastern Finland, Kuopio, Finland; 7 Oral Health Services, City of Tampere, Finland; 8 Neurosurgery, Tampere University Hospital, Tampere, Finland; 9 Department of Neurosurgery, Tampere University, Tampere, Finland

**Keywords:** Intracranial aneurysm, Subarachnoid hemorrhage, Caries, Periodontitis, Oral disease, Inflammation

## Abstract

**Purpose:**

Periodontal diseases and caries are common oral diseases that predispose to tooth loss if untreated. In this study, we investigated whether loss of teeth or caries associate with intracranial aneurysm (IA) pathology similar to periodontal diseases.

**Methods:**

A total of 166 patients with either IA or aneurysmal subarachnoid hemorrhage (aSAH) underwent oral examination in Kuopio University Hospital and Tampere University Hospital. Findings were compared to geographically matched controls acquired from cross-sectional Health2000 survey. This study consisted of three sequential steps. First, we compared the number of missing teeth and prevalence of caries in IA and aSAH patients and geographically matched control population, second step was a multivariate analysis including other risk factors, and third step was a 13-year follow-up of the Health2000 survey participants with missing teeth or caries at baseline.

**Results:**

Loss of teeth did not significantly differ between IA patients and controls. In logistic regression model adjusted for known risk factors and demographic data, 1–4 caries lesions (OR: 0.40 95%Cl 0.2–0.9, *p* = 0.031) was associated with lack of IAs, while age (OR: 1.03 95%Cl 1.01.1 *p* = 0.024), current smoking (OR: 2.7 95%Cl 1.4–5.1, *p* = 0.003), and severe periodontitis (OR: 5.99 95%Cl 2.6–13.8, *p* < 0.001) associated to IA formation. In the cox-regression, severe periodontitis at baseline increased the risk of aSAH (HR: 14.3, 95%Cl 1.5–135.9, *p* = 0.020) during a 13-year follow-up, while caries or missing teeth did not.

**Conclusion:**

Unlike severe periodontitis, caries does not increase the risk of IAs and aSAHs. However, cariogenic bacteria may participate to IA pathology by disseminating to circulation via inflamed gingival tissue.

**Supplementary information:**

The online version contains supplementary material available at 10.1007/s00701-022-05406-4.

## Introduction

Unruptured intracranial aneurysms (UIA) are frequent: They are found in approximately 3% of the past middle age population as mostly asymptomatic cerebral artery lesions [20]. Despite the rather high prevalence of UIAs, intracranial hemorrhage resulting from IA rupture is a relatively rare event (incidence approximately 10/100 000 in most countries) [14]. This form of intracranial hemorrhage known as aneurysmal subarachnoid hemorrhage (aSAH) has, however, a particularly sinister prognosis with a mortality reaching almost 50% and with many of the survivors left significantly disabled [18]. The best treatment for aSAH is to prevent it, a goal for which it is paramount to predict which UIA is going to eventually rupture and who will develop IAs in the first place.

Chronic inflammation or inflammatory cell mediated remodeling of the cerebral artery has been shown to be a crucial mediator of IA formation, as well as of the IA wall degeneration that eventually leads to rupture [2–4, 9]. The presence of bacterial derived DNA in the IA walls, and the expression of toll-like receptors that react to bacterial components and activate the immune system has been shown earlier [17]. This strongly implies that dental pathogens play a role in the inflammation mediated artery and aneurysm wall remodeling that leads to IA formation and aSAH. In addition, a prior study using RT-qPCR demonstrated that this bacterial DNA originates from several dental pathogens [17], consequently suggesting possible association of several oral diseases with IAs.

*Streptococcus mutans* is a classical dental pathogen involved in caries, a highly prevalent dental infection that leads to formation of cavities in teeth and, if untreated, to inflammation and necrosis of the tooth’s neural and vascular system, the tooth pulp. Eventually, these conditions may lead to root canal treatment or extraction of the affected tooth, i.e., tooth loss. Certain *S. mutans* strains like the Cnm and Cbm possess proteins capable of binding collagen and are linked to aggressive form of caries [12]. *S. mutans* expressing collagen-binding protein (CBP) has been previously shown to be more prevalent in oral samples of cerebral hemorrhage patients [13] and the persons affected by aSAH or other forms of intracranial hemorrhage [8]. CBP of *S. mutans* has been shown in animal models to predispose to intracranial hemorrhage through affecting the cerebral artery wall directly and can be detected in cerebral hemorrhage tissue after oral administration [13]. It seems possible that this pathogen, so important in caries, would also play role in IA formation and rupture.

It was recently observed that gingivitis and periodontitis*,* i.e., chronic infection of the gums and tooth supporting tissues, associates with the risk of developing IAs and eventually aSAH [6, 16]. If left untreated, periodontitis eventually predisposes and leads to loss of teeth. Before that, if the periodontitis is severe enough, the inflamed gingival tissue can act as a gateway for oral bacteria to enter the bloodstream [7, 19] and spread in the vasculature.

Since prior literature suggest the involvement of the caries pathogen *S. mutans* in IA formation and rupture and prior studies on the bacterial DNA present in IA wall tissue suggesting that both periodontal or cariogenic pathogens are found in the IA wall [17], we investigated whether caries in addition to periodontitis is associated with the risk of IA formation or rupture.

## Material and methods

This study was performed in three sequential steps. Because IAs develop overtime and prolonged exposure to untreated caries or periodontitis eventually leads to tooth loss, in the first step, we investigated whether the number of missing teeth associated with IA formation (UIA) or rupture (aSAH). This was done by comparing the number of missing teeth between IA and aSAH patients and the Health 2000 survey participants that formed our control group. Following this, we compared the prevalence of the two most common causes of tooth loss, i.e., caries and periodontitis among UIA and aSAH patients and the control group. The second step was a multivariate analysis of factors associated with UIA formation or aSAH, including missing teeth, caries, periodontitis, smoking status, and demographic data as the studied variables. In the third step, we studied in follow-up cohort of Health survey participants, whether caries or number of missing teeth at baseline would associate with aSAH during a 13-year follow-up.

This study was approved by the ethical review boards of the Healthcare District of Northern Savo, Pirkanmaa Hospital District, and The Ethical Committee for Research in Epidemiology and Public Health at the Hospital District of Helsinki and Uusimaa in Finland (the Health 2000 and the Health 2011 Surveys). Written informed consent was obtained from all participants.

### Study population for the case series of IA patients

Patients referred to the Departments of Neurosurgery of Kuopio university hospital (KUH) and of Tampere university hospital (TaUH) for IA treatment were recruited to this study. A detailed description of the formation of the study cohorts is given in the data supplement. A clinical oral examination was performed on 76 IA patients in KUH (42 with unruptured IAs and 34 with aSAH) and for 90 IA patients in TaUH (60 with unruptured IAs and 30 with aSAH). Patient selection was random due to the limited availability of an examining dentists (JH and MP), and interexaminer validation was not done. In KUH IA patients, caries lesions were common, but caries diagnostics could not be done due to the lack of panoramic X-rays, and KUH IA patients were therefore not included in other analysis besides that focusing on the number of missing teeth, as detected in the clinical oral examination. In TaUH IA patients, a number of teeth and dentin caries lesions were examined from panoramic X-ray. Caries was diagnosed when the caries lesion was clearly extending to dentin (a bone-like matrix under tooth enamel). We further categorized participants according to no caries lesions in teeth, 1–4 caries lesions, or > 5 caries lesions in teeth. Periodontitis was diagnosed taking into account the deepest periodontal probing depth, categorized as follows: < 4 mm no periodontitis, 4–5 mm periodontitis, and ≥ 6 mm severe periodontitis [6, 16]. For the KUH and TaUH IA patients, clinical data including known risk factors (age, gender, and current smoking) was collected from the medical reports and a personal interview.

### Case–control comparison of IA patients with the Health 2000 survey participants

Control group was formed from a previously published prospectively collected cohort of 8028 adult Finns of whom a total of 5144 participants underwent a baseline oral and periodontal examination. From this group of 5144 participants, we selected the geographically matched controls from TaUH area (*n* = 340) as our control group for the studied IA and aSAH patients of Tampere University Hospital. Demographics and risk factors for IA were collected using a questionnaire as described previously [1]. To identify possible prior UIA or aSAH before baseline, the national registry for hospital discharge diagnosis (HILMO) was searched for ICD-10 codes I67.1 or I60.0–I60.9. Also, procedure codes were searched for surgical or endovascular procedures according to the Nordic Classification for Surgical Procedures.

### Prospective follow-up of the Health 2000 survey participants

Follow-up data for all the 5144 Health 2000 survey participants that underwent clinical oral examination were collected from HILMO registry after the baseline examination but before December 31 2013 as previously described [6]. Patients were identified by having both ICD10 and procedure code relevant to aSAH. Cox regression was used to calculate the hazard ratio for IA formation and for aSAH during the 13-year follow-up after the baseline examination.

### Statistical analyses

Data was presented as frequencies or medians with ranges. Fisher’s exact test or chi-square tests were used to compare categorical variables and Mann–Whitney *U* test for continuous variables. Multivariate logistic regression and Cox regression were performed as described above. Results from both regression analyses were presented as odds or hazard ratios with 95% confidence intervals. SPSS 22.0 statistical software (IBM) was used, and a *P* value < 0.05 was considered as significant.

## Results

### Missing teeth and prevalence of caries among UIA and aSAH patients

As the first step, we investigated whether loss of teeth, i.e., number of missing teeth, associated with the formation of IAs or aSAH from an intracranial aneurysm. UIA patients from both KUH and TaUH cohorts had a median of only 2 missing teeth, and aSAH patients had median of 1 missing teeth, whereas in the control population, the number of missing teeth was 1 (Tables 1 and 2). After dividing the study group to quartiles according to the number of missing teeth, there was a trend towards higher number of missing teeth among UIA and aSAH patients compared to the control population, but this trend remained non-significant (data not shown).

**Table 1:** Demographics, dentin caries and number of missing teeth of TAUH unruptured IA (UIA) patients and geographically matched controls. Patients with SAH excluded from analysis

Variable	UIA patients (*n* = 60)	Matched controls (*n* = 340)	*P* value
Age	57.0 (23.0–74.0)	48.0 (30.0–89.0)	0.019*
Gender (number of females)	43/60 (71.7%)	207/340 (60.9%)	0.065
Current smoking	29/60 (48.3%)	104/339 (30.7%)	0.002*
At least one dentin caries	11/60 (18.3%)	87/340 (25.6%)	0.392
No caries	49/60 (81.7%)	251/340 (73.8%)	0.724
Caries in 1–4 teeth	10/60 (16.7%)	79/340 (23.2%)	0.724
Caries in > 5 teeth	1/60 (1.7%)	10/340 (2.9%)	0.724
Median of missing teeth	2 (0 –28)	1 (0–27)	0.956
Missing teeth divided to quartiles			
0–7 missing teeth	49/60 (81.7%)	281/340 (82.6%)	0.963
8–14 missing teeth	5/60 (8.3%)	25/340 (7.4%)	0.963
15–21 missing teeth	4/60 (6.7%)	16/340 (4.7%)	0.963
22–28 missing teeth	2/60 (3.3%)	18/340 (5.3%)	0.963
Periodontitis	47/60 (78.3%)	184/340 (54.1%)	< 0.001*
Severe periodontitis	24/60 (40.0%)	51/340 (15.0%)	< 0.001*

Data presented as median and range or as proportions. *P* values were calculated with Fisher’s exact test and with Mann–Whitney *U* test for continuous variables respectively

**Table 2:** Demographics, dentin caries and number of missing teeth of TAUH aSAH patients and geographically matched controls. UIA patients excluded from analysis

Variable	aSAH patients (*n* = 30)	Controls (*n* = 340)	*P* value
Age	49.0 (31.0–66.0)	48.0 (30.0–89.0)	0.107
Gender (number of females)	14/30 (46.7%)	206/340 (60.6%)	0.133
Current smoking	16/30 (53.3%)	104/340 (30.6%)	0.011*
At least one dentin caries	9/30 (30.0%)	87/340 (25.6%)	0.591
Median of missing teeth	1 (0–26)	1 (0–27)	0.863
No caries	21/30 (70.0%)	251/340 (73.8%)	0.724
Caries in 1–4 teeth	8/30 (26.7%)	79/340 (23.2%)	0.724
Caries in > 5 teeth	1/30 (3.3%)	10/340 (2.9%)	0.724
Missing teeth divided to quartiles			
0–7 missing teeth	24/30 (80.0%)	281/340 (82.7%)	0.871
8–14 missing teeth	3/30 (10.0%)	25/340 (7.4%)	0.871
15–21 missing teeth	2/30 (6.7%)	16/340 (4.7%)	0.871
22–28 missing teeth	1/30 (3.3%)	18/340 (5.3%)	0.871
Periodontitis	23/30 (76.7%)	184/340 (54.1%)	< 0.001*
Severe periodontitis	14/30 (46.7%)	51/340 (15.0%)	< 0.001*

Data presented as median and range or as proportions. *P* values were calculated with Fisher’s exact test and with Mann–Whitney U test for continuous variables respectively

Next, we investigated whether this trend of missing teeth among UIA or aSAH patients was related to severe periodontitis previously associated with IA disease, or whether caries, the other common cause of tooth loss is a more significant cause for it. Since data on both the periodontal status as well as caries was available only in the TAUH cohort, we focused our analysis on only this cohort. At least one dentin caries lesion was found in 18.3% of the UIA patients (Table 1) and 30.0% of the aSAH patients (Table 2). A 25.6% of the controls from the same region had at least one dentin caries lesion (Tables 1 and 2), and the difference was not statistically significant. There were no significant differences in subgroups categorized by number of caries lesion as shown above (Tables 1 and 2).

### Periodontitis but not caries associates with increased risk of IA formation and aSAH

After adjusting the logistic regression model for age, gender, and current smoking, presence of 1–4 dentin caries lesions had a negative association with IA formation (OR: 0.4 95%Cl 0.2–0.9, *p* = 0.031, Table 3, model 1). In a model including dentin caries categories, severe periodontitis remains independently associated with UIA and aSAH (OR 4.3, 95%Cl 2.3–12.5, *p* < 0.001; and OR 5.4, 95%Cl 1.9–15.5, *p* = 0.002, respectively, Table 3, models 1 and 2). The number of missing teeth was not associated with UIAs nor aSAH (Table 3 models 1 and 2).

**Table 3:** Association of dentin caries and number of missing teeth to IA formation and aSAH with periodontitis in a multivariate analysis comparing TAUH IA patients with a geographically matched control group of the Health 2000 participants

Variable	Odds ratio	95% CI	*P* value
IA formation (model 1) (60 unruptured IA cases in analysis)			
Age	**1.03**	1.004–1.061	0.024*
Gender (number of males)	**1.73**	0.885–3.373	0.109
Current smoking	**2.68**	1.393–5.134	0.003*
No caries	**1**		
Caries in 1–4 teeth	**0.40**	0.174–0.920	0.031*
Caries in > 5 teeth	**0.23**	0.027–2.012	0.128
One or more missing teeth (wisdom teeth excluded)	**0.858**	0.416–1.772	0.680
Periodontitis Severe periodontitis	**2.15** **5.99**	1.021–4.539 2.603–13.763	0.044* < 0.001*
aSAH (model 2) (30 aSAH cases in analysis)			
Age	**1.01**	0.968–1.044	0.775
Gender (number of males)	**0.70**	0.307–1.591	0.393
Current smoking	**2.17**	0.907–5.189	0.082
No caries	**1**		
Caries in 1–4 teeth	**0.72**	0.281–1.861	0.501
Caries in > 5 teeth	**0.55**	0.060–4.956	0.591
One or more missing teeth (wisdom teeth excluded)	**0.80**	0.310–2.046	0.637
Periodontitis Severe periodontitis	**1.41** **5.62**	0.490–4.040 1.946–16.228	0.526 0.001*

### Unlike severe periodontitis, caries did not associate with later aSAH in a 13-year follow-up

In a Cox-regression model adjusted for age, gender, current smoking, caries, periodontitis, and missing teeth at baseline, only severe periodontitis at baseline increased the risk of aSAH (Table 4).

**Table 4:** Risk of aSAH during a 13-year follow-up. COX regression model of the Health 2000 participants

Variable	Hazard ratio	95% CI	*P* value
aSAH (8 events in analysis)			
Age	**1.00**	0.920–1.077	0.904
Gender (number of males)	**3.76**	0.737–19.163	0.111
Current smoking	**4.03**	0.877–18.519	0.073
No caries	**1**		
Caries in 1–4 teeth	**0.35**	0.041–2.876	0.325
Caries in > 5 teeth	**None in analysis**		
One or more missing teeth (wisdom teeth excluded)	**0.56**	0.110–2.899	0.493
Periodontitis Severe periodontitis	**1.90** **14.31**	0.170–21.210 1.507–135.864	0.602 0.020*

## Discussion

IAs develop overtime within a variable timeframe. Caries and periodontitis are diseases that often stay chronic, until sufficient intervention is performed. If left untreated, progression of caries or chronic periodontitis eventually leads to tooth loss. Thus, in this study, we investigated, in addition to currently detected caries or periodontitis, also the number of missing teeth as a sign of prior exposure to progressed caries or periodontitis.

In this study, caries associated inversely with the formation of IAs, which probably reflects the fact that when caries becomes symptomatic, it leads to dental care and subsequently also concomitant treatment of other oral diseases, like periodontitis. Caries is the most common disease globally affecting nearly 3.5 billion people worldwide [10]. Even though caries is more prevalent in high-socioeconomic states [10], our study of high-socioeconomic population did not associate caries to a greater risk of IA and aSAH. There are several explanations for the lack of association: First, the nature of caries is a local infectious disease of the tooth structure with systemic responses only when the caries reaches the tooth pulp. Secondly, caries is most often treated with fillings before severe conditions, such as inflammation of tooth pulp or periapical abscesses occur. Thirdly, caries does not predispose to bacteremia contrary to periodontal diseases of the gingival tissues.

The association studies of oral bacteria with vascular diseases, including sIA/aSAH concentrate mostly on periodontal bacteria. Regarding cariogenic bacteria, a Japanese study [8] linked specific cnm strain of *Streptococcus mutans* to sIA. This specific strain promotes platelet aggregation inhibition and matrix metalloproteinase-9 activation which can logically be linked with sIA formation and rupturing. Although in our study, caries, nor the number of caries lesions, did not associate with sIA disease; the finding of Inenaga and colleagues may be explained by the insufficient oral hygiene predisposing to a poorer periodontal status. Via inflamed periodontal tissues, this specific *S. mutans* strain could disseminate to circulation and to IA walls. This explanation is, however, highly speculative.

### Other dental infections as risk factors for IA formation and subsequent aSAH

Whereas caries does not appear to associate with IA and aSAH, gingivitis and periodontitis, however, seem to associate to both IAs and aSAH [5, 6, 16]. This study served as replication study in a separate independent study cohort to our prior finding of the association between severe periodontitis and IA disease [4]. Similar association of periodontitis to IAs and aSAHs that we have previously reported in the KUH cohort [6] was also clear in this study with TaUH patient cohort when IA patients were compared with geographically matched healthy controls. Previously, a plethora of studies have associated periodontitis to cardiovascular diseases [11, 15], which demonstrates the crucial role of gingival pocket health as the border between systemic circulation and oral cavity. The pathomechanisms of caries are not similar with that of periodontal diseases.

Since periodontal diseases associate with sIA disease and increase the risk of aSAH in the long term, but other oral diseases, to our knowledge, do not, the effect and association might be explained by the nature of the disease itself rather than periodontal disease being only an “innocent co-variant” reflecting for example poor socioeconomic status to which many IA risk factors associate to. A prior study on the bacterial DNA present in IA wall tissue has not been able to discriminate whether periodontal or cariogenic pathogens associate with IAs, but rather DNA of pathogens related to both groups are found in the IA wall [17]. This discrepancy between the bacterial DNA findings and the clinical finding that nevertheless only periodontal disease associates with IA formation or rupture could be explained by the bleeding wound surface in inflamed gingival pocket forming a gateway for oral bacteria to enter the systemic circulation and eventually the IA wall. Further studies on this hypothesis are warranted.

## Limitations of the study

Our study has some limitations due to its design. First, we could not recruit all IA and aSAH patients to the study due to patient’s denial to participate or due to tight schedule of the examining dentists. Because of this, our study cohort represents only a subcohort of all the IA patients that were treated at TaUH or KUH during the recruitment period. In spite of this, our study cohort has the necessary statistical power to detect a possible association between caries and IA formation or rupture, since the same cohort shows similar association with periodontitis. Thus, we can conclude that even if one would question the statistical power of our study cohort and suspect that there might still be an association between caries and IA disease that we just could not detect in our study, it is clear of the oral diseases that periodontitis is many times more significantly associated with IA formation or rupture than caries. Of note is that in our study, caries was actually associated with a reduced risk of IA formation, which likely reflects the fact that caries leads to dental care which often improves also periodontal disease. Nevertheless, this observation makes type II error in our study highly unlikely.

The use of healthcare registry data has always a source of bias. Also, our comparison between KUH and TaUH patients and the Health2000 survey participants has its limits, since there is a 15–16-year gap between oral examinations. Also, single person performing the oral examination (JH in KUH and MP in TaUH) could lead to over- or underestimations of oral conditions.

## Conclusion

Unlike severe periodontitis, caries does not associate with the IA formation or aSAH. However, cariogenic bacteria may participate to IA pathogenesis, but further research is needed.

## Supplementary Information

Below is the link to the electronic supplementary material.

Supplementary file1 (DOCX 29 KB)
