# Supplementary material for: Unlike severe periodontitis, caries does not associate with intracranial aneurysms or aneurysmal subarachnoid hemorrhage
Source: Acta Neurochir (Wien). 2022 Nov 23;165(1):169–75. doi: 10.1007/s00701-022-05406-4 (PMC9840572; doi:10.1007/s00701-022-05406-4)
Supplement: Supplementary file 1 — Supplementary file1 (DOCX 29 KB) [file 701_2022_5406_MOESM1_ESM.docx]

..
